# Evaluating the Toxic Effects of Tannic Acid Treatment on *Hyphantria cunea* Larvae

**DOI:** 10.3390/insects13100872

**Published:** 2022-09-26

**Authors:** Mingtao Tan, Hongfei Wu, Shanchun Yan, Dun Jiang

**Affiliations:** 1 School of Forestry, Northeast Forestry University, Harbin 150040, China; 2 Key Laboratory of Sustainable Forest Ecosystem Management-Ministry of Education, Northeast Forestry University, Harbin 150040, China

**Keywords:** tannic acid, *Hyphantria cunea*, detoxification, oxidative stress, gut microbiota

## Abstract

**Simple Summary:**

The high concentration of tannic acid in the needles of larch, a non-host plant, was inferred to be the root cause of the antifeedant activity displayed by *Hyphantria cunea* towards larch needles. In this study, tannic acid treatment was found to significantly increase the mortality and inhibit the body weight and food utilization of *H. cunea* larvae. Furthermore, an increase in the reactive oxygen species content and a decrease in antioxidant and detoxification enzyme activities in midgut tissues, as well as disorders in the gut microbiota, were observed in tannic acid-treated larvae. The findings reveal that tannic acid can be used as a plant-derived pesticide for the green prevention of *H. cunea* larvae.

**Abstract:**

To increase the development potential of botanical pesticides, it is necessary to expand the toxicology research on plant secondary metabolites. Herein, the *Hyphantria cunea* larvae were exposed to tannic acid concentrations consistent with those found in larch needles, and, subsequently, the growth and nutrient utilization, oxidative damage, and detoxification abilities in the larval midgut, as well as the changes in the gut microbiome, were analyzed. Our results revealed that tannic acid treatment significantly increased the mortality of *H. cunea* larvae and inhibited larval growth and food utilization. The contents of malondialdehyde and hydrogen peroxide in the larval midgut were significantly elevated in the treatment group, along with a significant decrease in the activities of antioxidant enzymes and detoxifying enzymes. However, the non-enzymatic antioxidants showed a significant increase in the tannic acid-treated larvae. From gut microbiome analysis in the treatment group, the abundance of gut microbiota related to toxin degradation and nutrient metabolism was significantly reduced, and the enrichment analysis also suggested that all pathways related to nutritional and detoxification metabolism were substantially inhibited. Taken together, tannic acid exerts toxic effects on *H. cunea* larvae at multiple levels and is a potential botanical pesticide for the control of *H. cunea* larvae.

## 1. Introduction

Plants and insects have interacted and co-evolved with each other for more than 350 million years. Secondary metabolites, as the end products or by-products of plant metabolism, are important mediums for the co-evolution of phytophagous insects and their host plants [1]. At present, nearly 400,000 secondary metabolites are known to be synthesized by plants, and these can be mainly divided into phenols, terpenoids, and nitrogen-containing compounds [2]. Among these, some secondary metabolites exert adverse effects on insects, including antifeedant, repellent, or toxic effects [3,4]. Common secondary metabolites with toxic effects include flavonoids and phenols, among others [3,4,5]. For example, Sun et al. report that artificial diets containing gossypol, tomatine, nicotine, or capsaicin could significantly inhibit the feeding of *Helicoverpa armigera* larvae (Lepidoptera: Noctuidae) [5]. Chen et al. show that feed supplemented with quercetin can directly hinder the normal growth and development of *H. armigera* larvae [6]. In general, plant secondary metabolites are an important weapon against phytophagous insects; plants synthesize a large number of defensive secondary metabolites by inducing a defense response when threatened by insects [7,8,9]. This has been reported in several plants, such as corn and tomato [7,8]. In addition, plants can also synthesize some volatile secondary metabolites, thereby exerting attractive effects. These volatile compounds can lure predatory or parasitic natural enemies to indirectly control pests [10,11]. 

To resist the defensive metabolites produced by host plants, phytophagous insects also activate their defense mechanisms (e.g., anti-feeding, reducing feeding, activating detoxification, and antioxidant defense systems) to maintain their populations [12,13]. Among these, the antioxidant defense system composed of antioxidant enzymes (such as peroxidase (POD), catalase (CAT), and superoxide dismutase (SOD)), and non-enzymatic antioxidants (such as ascorbic acid (ASA) and glutathione (GSH)), is known to alleviate reactive oxygen species (ROS) outbreak or oxidative damage induced by plant secondary metabolites [14]. This is the main mechanism underlying insect resistance to plant secondary metabolites [15]. In addition, the detoxification mechanism based on detoxification enzymes also plays an important role in the metabolism and adaptation of insects to plant secondary metabolites and is thus another mechanism of resistance in phytophagous insects against secondary metabolites of the host plant [13,16]. The enzymes involved in the metabolism and detoxification of toxicants in insects mainly include cytochrome P450 enzymes (P450), carboxylesterase (CarE), and glutathione S-transferase (GSTs). For example, as demonstrated by Zhang et al., the *Bombyx mori* (Lepidoptera: Bombycidae) larvae can resist the toxicity of quercetin by increasing the activities of P450, GSTs, and CarE [17]. In addition to the physiological defense mechanisms composed of detoxification and antioxidant systems, in recent years, it has been reported that the gut microbiota of phytophagous insects also provides an important defense against detoxifying secondary metabolites. The gut of insects provides a suitable environment for the colonization of several microbes, which, in turn, provide many potential benefits to their host insects, including assisting in metabolism, enhancing immunity and environmental adaptability, and improving defense and detoxification [18,19,20,21,22].

*Hyphantria cunea* (Lepidoptera: Erebidae), also known as the fall webworm, is a quarantine pest found worldwide [23]. In the past few decades, *H. cunea* has spread from North America to many countries in Asia, Europe, and the Americas through anthropogenic activities. Owing to the characteristics of *H. cunea*, such as miscellaneous food habits, high reproduction, and fast transmission, grave economic losses to agroforestry production in the areas of an outbreak have occurred [24,25,26]. The crops and forest plants that the larvae of *H. cunea* often feed on include hickory, pecan, walnut, elm, alder, willow, mulberry, oak, sweetgum, and poplar [27]. At present, several control strategies have been adopted to reduce the harm caused by *H. cunea.* However, these prevention and control strategies practiced in different countries mainly involve chemical prevention. Given the fact that the large-scale use of chemical pesticides easily results in the “Resistance, Resurgence and Residue” phenomenon and serious pollution of the ecological environment, there is an urgent demand for a substitute to slow down the use of chemical pesticides [28]. Relative to traditional chemical pesticides, botanical pesticides have the characteristics of high safety, environmental friendliness, and low residues, thus endowing them with the potential to replace chemical pesticides used in the control of *H. cunea* [29,30,31]. Plant secondary metabolites exert toxic effects on insects and can be more easily degradable in the environment and, thus, are important candidates for the synthesis of botanical pesticides [32]. To reduce the use of chemical pesticides and develop environmentally friendly botanical pesticides, it is necessary to expand the knowledge on the toxic effects of plant secondary metabolites on *H. cunea* and their underlying mechanism.

As a common plant secondary substance, tannins can be divided into hydrolyzable tannins (HTs) and concentrated tannins (CTs) [33]. Tannin reportedly exerts toxic effects on many phytophagous insects, including *Oedaleus asiaticus* (Orthoptera: Oedipodidae), *Choristoneura fumiferana* (Lepidoptera: Tortricidae), and *Halyomorpha halys* (Hemiptera: Pentatomidae) [34,35,36]. It has a broad application prospect in the green control of phytophagous pests and is a potential botanical pesticide. In our previous study, we found that *Larix olgensis* (Pinales: Pinaceae) is a non-feeding host of *H. cunea* larvae, and it can result in the production of significant antifeedant effects by the larvae (unpublished). Further analysis showed that *L. olgensis* needles contain high concentrations of tannic acid [37,38]. Therefore, we reasonably speculated that the high concentration of tannic acid in needles was the root cause for the antifeedant activity of *H. cunea* towards larch needles and that tannic acid could be used for the green control of *H. cunea* larvae. In this study, *H. cunea* larvae were exposed to tannic acid concentrations consistent with those found in the needles of larch, a non-host plant. Subsequently, the growth and nutrient utilization, oxidative damage, and detoxification abilities in the midgut, as well as gut microbiome alterations in the larvae, were analyzed. The relevant findings may help us better understand the toxic effects and the mechanism of tannic acid action on *H. cunea* larvae, thus laying a theoretical foundation for follow-up research and development for the application of tannin-based botanical pesticides.

## 2. Materials and Methods

### 2.1. Insect Rearing

The egg masses of *H. cunea* and artificial diets were purchased from the Chinese Academy of Forestry Sciences (Beijing, China) in July 2019. The eggs were disinfected in 10% formaldehyde solution and hatched in a light incubator with a relative humidity of 70 ± 5% at a temperature of 25 ± 1 °C and photoperiod of 16L:8D. The newly hatched larvae were fed on an artificial diet till the 3rd instar stage, under the original conditions. 

### 2.2. Tannic Acid Treatment

Tannic acid (purity: 98%; CAS: 1401-55-4) was purchased from Shanghai Macklin Biochemical Co., Ltd. The tannic acid concentration was set at 12.5 mg/g, within the range of the tannic acid concentration in *L. olgensis* needles (4.17–72.07 mg/g) [37,38]. Tannic acid was dissolved with distilled water (250 mg/mL), and then 10 mL of tannic acid solution was added to 190 mg artificial diets to prepare a diet with a tannic acid concentration of 12.5 mg/g. In the untreated artificial diets, we added 10 mL of distilled water. Subsequently, the healthy larvae at 3rd instar were divided into two groups: one fed on an untreated artificial diet (control group) and the other on a tannic acid-treated artificial diet (treatment group). The artificial diet was changed once a day until the larvae pupated. A total of 300 larvae were raised in each group. Of these, 150 larvae were used to calculate mortality, and the remaining larvae were used for subsequent experiments. In the experiment to calculate mortality, we set up three replicates, each of which included 50 *H. cunea* larvae.

### 2.3. Determination of Mortality, Growth, and Nutrition Utilization

During the experiment, the larval mortality, pupation rate, and emergence rate of the larvae in the control and treatment groups were calculated. The 5th instar larvae that were about to molt in each group were picked out. After molting to 6th instar, 30 larvae in each group were weighed. Subsequently, these 6th instar larvae were fed on the untreated or tannic acid-treated artificial diets for 24 h, and the nutrient utilization was determined according to the method described by Jiang et al. [14]. A total of thirty repeats in each group were set, with one larva per repeat. Nutritional efficiency indexes, expressed as fresh weights, were calculated based on the following formulae:

Food intake = (fresh food weight before feeding − residual food weight after feeding)/ (1 − corrected water loss rate);

Approximate digestibility (AD) = (food intake − weight of feces)/food intake × 100%;

Efficiency of conversion of digested food (ECD) = larval weight gained/ (food intake − weight of feces) × 100%;

Efficiency of conversion of ingested food (ECI) = larval weight gained/food intake × 100%.

### 2.4. Determination of MDA and H_2_O_2_ Contents

The 6th instar larvae aged less than 24 h were dissected, and the excised midgut tissues were homogenized in pre-chilled saline. After centrifuging at 10,000× *g* (4 °C) for 10 min, the supernatants were collected and the levels of malondialdehyde (MDA) and hydrogen peroxide (H_2_O_2_) were detected using the corresponding kits (purchased from Nanjing Jiancheng Biology Co., Ltd., Nanjing, China). The assays were set in three repeats in each group, with five larvae per repeat. Briefly, H_2_O_2_ reacts with molybdic acid to form a complex, which can be spectrophotometrically detected at 405 nm. The H_2_O_2_ content was expressed in nmol/mg protein. MDA can react with thiobarbituric acid (TBA) to form a red product, which can be spectrophotometrically detected at 532 nm. The content of MDA was expressed in nmol/mg protein.

### 2.5. Determination of Antioxidant and Detoxification Capacities in the Larval Midgut

The 6th instar larvae aged less than 24 h were dissected, and the excised midgut tissues were homogenized in pre-chilled saline. After centrifuging at 3000× *g* (4 °C) for 10 min, the supernatants were collected and the activities or contents of CAT, POD, GSTs, ASA, and GSH were detected using the corresponding kits (purchased from Nanjing Jiancheng Biology Co., Ltd. (Nanjing, China)). A total of three repeats in each group were set, with five larvae per repeat. Specifically, CAT activity was detected by the ammonium molybdate method at 405 nm and expressed in U/mg protein. POD can catalyze H_2_O_2_, and its activity can be obtained by measuring the change in absorbance at 420 nm. The activity of POD was expressed in U/mg protein. GSTs can catalyze the binding of reduced glutathione (GSH) to the CDNB substrate. In the reaction system, the reduction of GSH by 1 μmol/L was a unit of enzyme activity, which could be detected at 412 nm. The GST activity was expressed in U/mg protein. ASA can react with Fe^3+^ to form Fe^2+^, which can further react with phenanthroline, resulting in color production. The reaction could be detected at 536 nm, and the content of ASA was expressed in μg/mg protein. The thio compounds in GSH can react with dithiodinitrobenzoic acid (DTNB), resulting in the formation of yellow compounds, which can be detected at 420 nm. The content of GSH was expressed in μg/mg protein. The protein content was determined by Coomassie brilliant blue staining at 595 nm.

### 2.6. Gut Microbiological Analysis

The 6th instar larvae aged less than 24 h were dissected, and the gut tissues of the larvae were obtained for microflora analysis. A total of four repeats in each group were set, with five larvae per repeat. The DNA of the gut flora in the *H. cunea* larvae was extracted using the E.Z.N.A. ^®^Stool kit and stored in a refrigerator at −80 °C until further use. After qualitative detection by 0.8% agarose gel electrophoresis, PCR was performed by Lianchuan Biotechnology Co., Ltd. (Hangzhou, China). The total DNA was used as a template for each sample, and the V3–V4 regions of 16S rDNA were amplified. The universal primer sequences were 341F (5′-CCTACGGGGNGGCWGCAG-3′) and 805R (5′-GACTACHVGGGGTATCTACC-3′). After the extracted 16S rRNA V3–V4 amplification products qualified the 2% gel electrophoresis analysis, the amplified sequences were sequenced using Ampre XT beads. The original sequence obtained was spliced by overlapping. An Agilent 2100 bioanalyzer and the Illumina quantitative kit were used to evaluate the sizes and numbers of the amplification sequences. The original data were screened for redundancy; the sequences with poor quality (less than 20%) and short sequences (<50 bp) were deleted. Subsequently, the chimeric sequences were filtered using VSearch (v2.3.4) to obtain high-quality sequences [39]. The clean sequences were obtained after de-repetition by DADA2 [40]. The alpha diversity (Chao1 and Shannon) was calculated using QIIME2 [41], while the beta diversity was calculated by principal coordinate analysis (PCoA) and principal component analysis (PCA). Linear discriminant analysis (LDA) effect size (LEfSe) was used to identify the microbial groups with differential relative abundances in the control and treatment groups.

### 2.7. Statistical Analysis

All the parameters of the control and treatment groups were analyzed and compared by an independent sample t-test with α = 0.05 as the significance level. The functions of microflora were predicted using PICRUSt2, and the relative abundances of gut microflora with the same functions between the control/treatment groups were compared by an independent sample t-test [42]. Based on the above physiological indexes and gut microflora data, Pearson’s correlation analysis was performed to determine the correlation between gut microorganisms at the genus level and the physiological parameters in tannic acid-treated larvae.

## 3. Results

### 3.1. Larval Mortality, Growth, and Food Utilization

**Figure 1:**
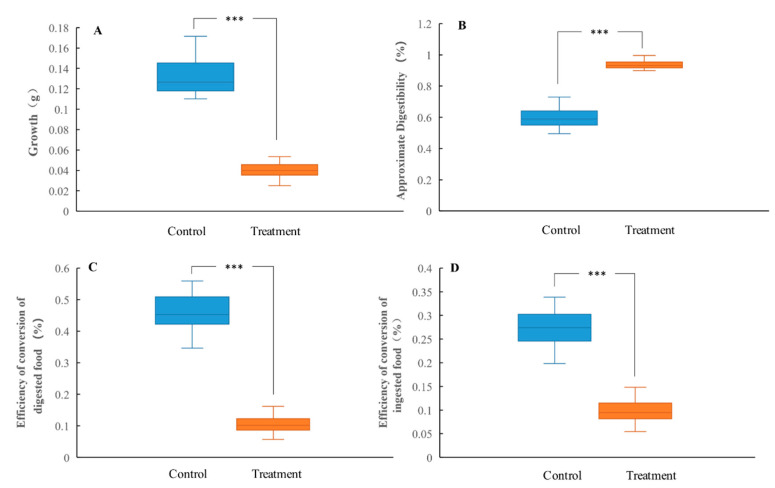
Effect of tannic acid on the growth (**A**), approximate digestibility (**B**), efficiency of conversion of digested food (**C**), and efficiency of conversion of ingested food (**D**) in the *H. cunea* larvae at the 6th instar. Asterisk indicates significant differences between control/treatment groups (*n* = 30; independent samples *t*-test; ***, *p* < 0.001).

To evaluate the toxic effects of tannic acid on *H. cunea* larvae, the larval mortality, pupation rate, emergence rate, and the growth (larval weight) and nutritional utilization indexes (AD, ECD, and ECI) of the 6th instar larvae in both the control and treatment groups were measured. As shown in Table 1, the cumulative mortality rate in the control group was 0% for the whole larval phase; however, under tannic acid treatment, the cumulative mortality of larvae was as high as 100%, accompanied by 6.67% cumulative mortality of 4th instar larvae, 19.33% cumulative mortality of 5th instar larvae, and 45.67% cumulative mortality of 6th instar larvae. In the control group, the pupation rate and emergence rate of *H. cunea* were 70% and 80.95%, respectively. However, the *H. cunea* in the treatment group did not pupate or emerge, and all died in the larval stage (Table 1). As shown in Figure 1A, the body weight of *H. cunea* larvae in the treatment group were significantly lower than those in the control group. After tannic acid treatment, the responses of ECD and ECI were consistent with the increasing trend, and both decreased significantly. However, tannic acid treatment significantly increased the AD of the larvae (Figure 1B–D).

### 3.2. Evaluation of Oxidative Damage in Midgut tissues

**Figure 2:**
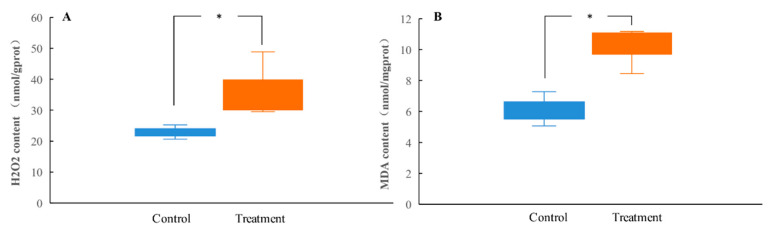
Effect of tannic acid on the H_2_O_2_ (**A**) and MDA (**B**) content in the *H. cunea* larvae at the 6th instar. Asterisk indicates significant differences between control/treatment groups (*n* = 3; independent samples *t*-test; *, *p* < 0.05).

To measure the degree of oxidative damage in the midgut tissues of the tannin-treated *H. cunea* larvae, the levels of H_2_O_2_ and MDA were measured. As shown in Figure 2A,B, the tannic acid treatment significantly enhanced the contents of H_2_O_2_ and MDA in the midgut of *H. cunea* larvae.

### 3.3. Larval Antioxidation and Detoxification Abilities

**Figure 3:**
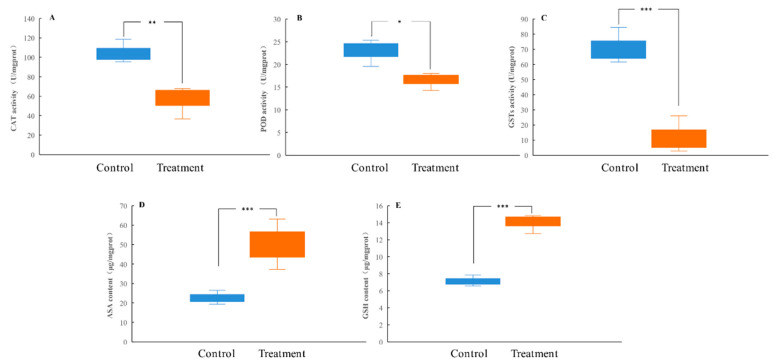
Effect of tannic acid on the activities of CAT (**A**), POD (**B**), and GSTs (**C**) and the content of ASA (**D**) and GSH (**E**) in the *H. cunea* larvae at the 6th instar. Asterisk indicates significant differences between control/treatment groups (*n* = 3; independent samples *t*-test; *, *p* < 0.05; **, *p* < 0.01; ***, *p* < 0.001).

To understand the defense mechanism of *H. cunea* larvae against tannic acid, the antioxidation and detoxification abilities in the larval midgut after tannic acid treatment were analyzed. The results showed that artificial diets amended with tannic acid were able to significantly decrease the activities of the antioxidant enzymes (CAT and POD) and detoxifying enzymes (GSTs) in the larval midgut while significantly increasing the levels of non-enzymatic antioxidants (ASA and GSH) (Figure 3A–E).

### 3.4. Analysis of the Microbial Diversity in the Larval Gut

**Figure 4:**
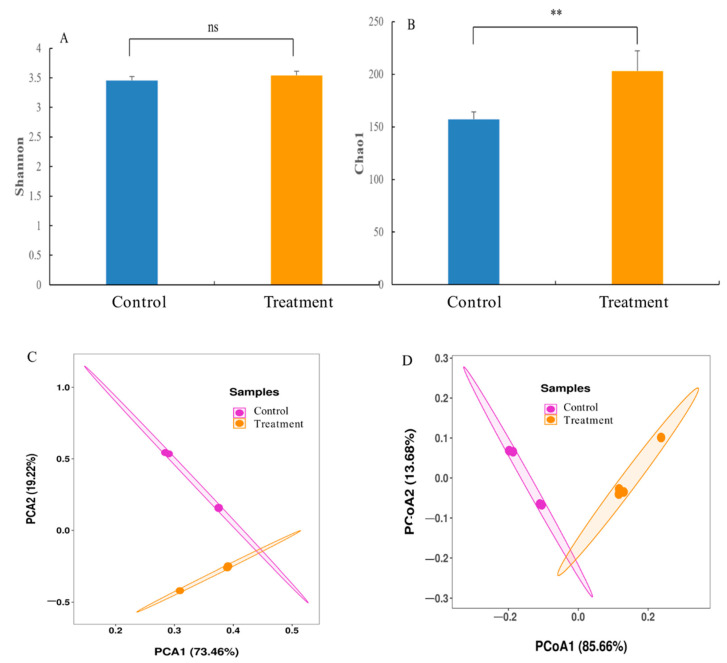
The gut microbial diversity in the *H. cunea* larvae at the 6th instar after rearing on untreated or tannic acid-treated diets (*n* = 4). (**A**) Chao1 index. (**B**) Shannon index. (**C**) Principal components analysis (PCA). (**D**) PCoA plot of the gut microbiota structures based on the unweighted UniFrac analysis. Asterisk indicates significant differences between control/treatment groups (independent samples *t*-test; **, *p* < 0.01).

Using 16S rRNA gene sequencing, a total of 582,959 high-quality transcripts were obtained from 8 samples, with an average of 72,870 transcripts. After classification, 771 operational taxonomic units (OTUs) were identified as having 97% sequence similarity. The Venn diagram showed 162 OTUs in the treatment and the control groups, while there were 399 and 210 specific OTUs in the treatment and the control groups, respectively (Figure S1). Subsequently, the α and β diversity indexes for microbial communities were evaluated and compared between the control/treatment groups. The results showed that the tannic acid treatment exerted no significant effects on the Shannon index of the gut microflora, but the Chao1 index in the tannic acid treatment group was found to be significantly high (Figure 4A,B). PCA showed that the gut flora of the larvae treated with tannic acid was well separated from that of the control group, and the principal components, PC1 and PC2, could explain 73.46% and 19.22% of the alterations, respectively (Figure 4C). Similarly, the PCoA analysis also showed that the composition of gut microflora in the tannic acid-treated group was significantly different from that in the control group (Figure 4D).

### 3.5. Taxonomic Composition of the Gut Microbiota

The taxonomic analysis of gut microbiota showed that all the sequences could be classified into 19 bacterial phyla. Among them, Proteobacteria was the dominant phylum of the larval microbial community, accounting for 92.91% and 77.76% of the total sequences in the control and treatment groups, respectively; Cyanobacteria was the second highest, accounting for 2.37% and 14.93%, respectively, followed by Firmicutes, accounting for 1.18% and 5.25%, respectively (Figure S2A). At the genus level, a total of 274 genera were identified from all 8 gut samples. The distribution proportion of the first 30 genera was sorted by their abundances, as shown in Figure S2B; a significant difference was observed between the control and treatment groups. The dominant genera were *Pseudomonas* (33.59%), *Brevundimonas* (27.64%), and *Delftia* (11.04%) in the control larvae, whereas, in the tannin-treated larvae, the dominant genera were *Pseudomonas* (37.43%), Mitochondria_unclassified (24.44%), and Chloroplast_unclassified (14.93%).

The LEfSe analysis showed that there were differences in the relative abundances of microflora between the control and treatment groups (Figure S3A,B). The larvae in the control group were mainly characterized by a high abundance of Actinobacteria and Proteobacteria, while those in the treatment group were characterized by a high abundance of Cyanobacteria and Firmicutes. The relative abundances of the 19 genera of microbiota were different between the control and treatment groups. The abundances of *Brevundimonas*, *Delftia*, *Caulobacter*, *Sphingomonas*, *Novosphingobium*, Actinobacteria_unclassified, *Patulibacter*, *Dechloromonas*, *Methyloversatilis*, *Pedobacter*, *Lutispora*, *Pir4_lineage,* and *Devosia* in the control group were significantly high, whereas those of Mitochondria_unclassified, *Hydrogenophaga*, Oxyphotobacteria_unclassified, *ZOR0006*, *Phenylobacterium*, and *Dongia* in the treatment group increased significantly (Figure S3A,B).

**Figure 5:**
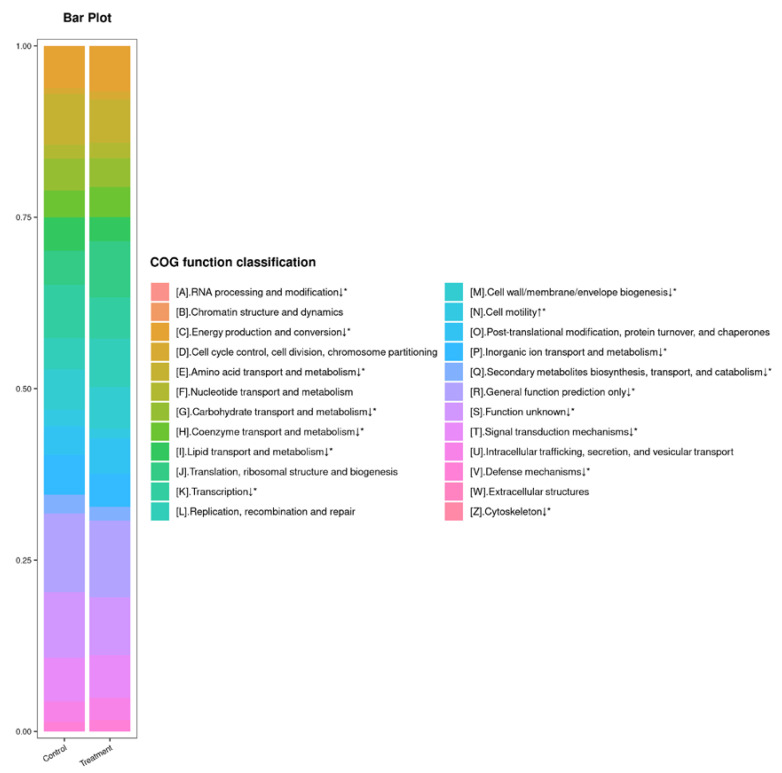
Summary of PICRUSt analysis. Asterisk indicates a significant difference in the relative abundances of the gut microbial communities (*n* = 4; independent samples *t*-test; *, *p* < 0.05).

To study the effect of tannic acid treatment on the functions of the gut microbiota, BLAST was used to predict the functions of the sequences by searching against the COG database. A total of 24 functional categories were annotated (Figure 5). Tannic acid exposure resulted in a significant upregulation of one functional category, namely, cell motility (*p* < 0.05). However, 14 functional categories, including RNA processing and modification; energy production and conversion; amino acid transport and metabolism; carbohydrate transport and metabolism; coenzyme transport and metabolism; lipid transport and metabolism; transcription; cell wall/membrane/envelope biogenesis; inorganic ion transport and metabolism; secondary metabolites biosynthesis, transport, and catabolism; general function prediction only; function unknown; signal transduction mechanisms; defense mechanisms; and cytoskeleton (*p* < 0.05), were significantly downregulated in the treatment group.

### 3.6. Correlation between Gut Microflora and Biochemical Parameters

**Figure 6:**
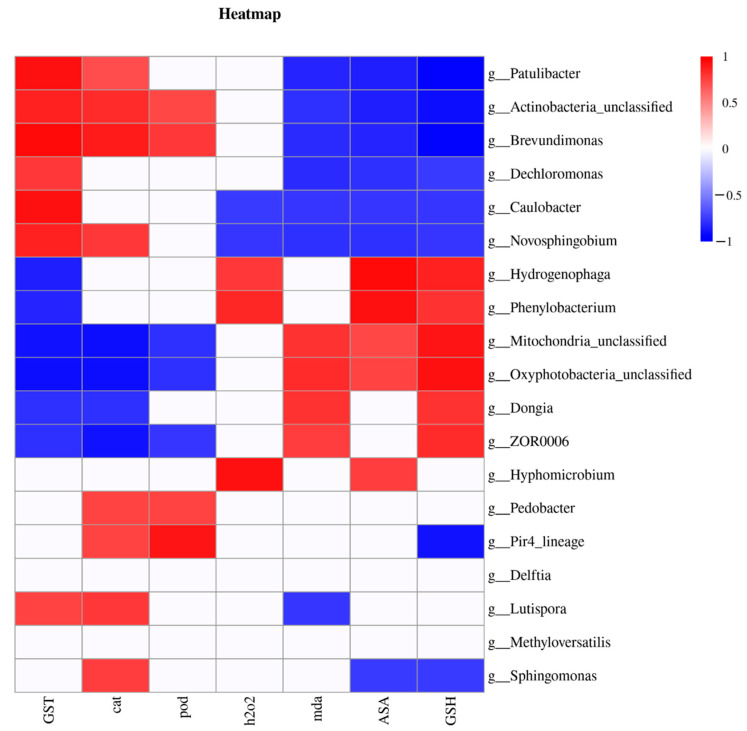
Correlation analysis of the larval gut microbiota (at the genus level) and physiological parameters in the *H. cunea*. Each square is filled with a color according to Pearson’s coefficient. Red represents a positive correlation (Z-test, *p* < 0.05); blue represents a negative correlation (Z-test, *p* < 0.05); and white represents a non-significant correlation (Z-test, *p* > 0.05).

To determine whether the gut microbes could affect the physiological state of the larval intestine, we analyzed the correlation between gut microflora at the genus level and the physiological parameters (Figure 6). Based on the Pearson correlation analysis, the differential gut microflora of the larvae could be divided into three types according to their correlations with biochemical parameters. The first type was positively correlated with antioxidant enzymes and detoxifying enzymes but negatively correlated with the non-enzymatic antioxidants and ROS content and was mainly composed of the following genera: *Patulibacter*, Actinobacteria_unclassified, *Brevundimonas*, *Caulobacter*, and *Novosphingobium*. In contrast, the second type was positively correlated with the non-enzymatic antioxidants and ROS levels but negatively correlated with antioxidant enzymes and detoxifying enzymes and mainly comprised the *Hydrogenophaga*, *Phenylobacterium*, Mitochondria_unclassified, Oxyphotobacteria_unclassified, *Dongia*, and *ZOR0006* genera. The third type consisted of the remaining seven genera that only showed correlations with a few physiological indexes.

## 4. Discussion

Several toxicological studies have shown that tannic acid is a plant secondary metabolite with insecticidal activity and can potentially serve as a raw material for developing botanical pesticides [34,35,36]. In this study, the toxicological effects of tannic acid on *H. cunea* larvae were systematically investigated by adding tannic acid to an artificial diet; additionally, the underlying mechanism was elucidated. The results showed that tannic acid exerted strong toxic effects on *H. cunea* larvae, with a 100% larval mortality rate. Tannic acid is a potential botanical pesticide that can be used for the control of *H. cunea* larvae.

As important indexes by which to measure the effects of toxic substances on insects [43], growth and food utilization can reflect the impact of tannic acid exposure on *H. cunea* larvae. In this study, tannic acid was found to significantly inhibit the growth and food utilization of *H. cunea* larvae. This may be attributed to the mechanism underlying the toxic effects of tannic acid. Tannic acid is an anti-nutritional factor that can adversely affect the feeding, growth, and development of phytophagous insects by interfering with the digestion of starch, lipid, and total protein in the gut [44,45,46]. A similar report by Yuan et al. investigated the effects of tannic acid on the growth and food utilization of *H. cunea*, using the treatment concentration of tannic acid equivalent to that in the host plant [43]. They demonstrated that tannic acid treatment at low dosages could promote the growth and food utilization of *H. cunea* larvae, but at high dosages, tannic acid treatment inhibited these parameters. Contrary to their research, the tannic acid concentrations were set in this study according to those in the non-host plants (with the aim of more accurately and systematically investigating the reason underlying the non-adaptation of *H. cunea* to non-host plants). Furthermore, the AD of *H. cunea* increased significantly in the tannin-treated group. AD is a parameter that measures the ability of phytophagous insects to assimilate food [47]. An increase in AD indicates that *H. cunea* larvae will employ feeding compensation measures for physiological adjustments to compensate for the problem of the low nutritional value of the tannin-treated artificial diet and to maintain normal biological processes. However, the increase in AD did not slow the decrease in ECD and ECI. These results clearly demonstrated that the digestive system of *H. cunea* larvae was dysregulated under tannic acid stress, which decreased the utilization of nutrients and led to their growth retardation. 

The ROS outbreaks and the disorder of the antioxidant defense system are important mechanisms by which exogenous stress factors, including plant secondary metabolites, exert toxic effects [48]. In this study, we measured the levels of ROS-related parameters (MDA and H_2_O_2_) to evaluate the degree of oxidative damage in the larval midgut. The results showed that the contents of MDA and H_2_O_2_ in the midgut increased significantly, thereby indicating that tannic acid induced ROS disorders and caused oxidative damage to the midgut tissues of *H. cunea* larvae. As the main site of digestion and absorption, the injury to midgut tissues affects its normal digestion and absorption functions [47]. This is consistent with the above results for growth and food utilization, which further proved the toxic effects of tannic acid on *H. cunea* larvae. To examine the mechanism underlying ROS disorders, the antioxidant defense systems, including the levels of the antioxidant enzymes and non-enzymatic antioxidants, were determined in this study. The results showed that the activities of antioxidant enzymes (e.g., CAT and POD) decreased significantly under tannic acid stress, thus indicating that tannic acid could inhibit the antioxidant ability of *H. cunea* larvae. This may possibly be the main reason for the outbreak and disorders of ROS in tannin-treated larval midguts. However, the contents of non-enzymatic antioxidants, an important component of the antioxidant defense system, increased significantly in the midgut of larvae treated with tannic acid, thus indicating that non-enzymatic antioxidants could not effectively alleviate tannic acid-induced ROS outbreak and replace the role of antioxidant enzymes. In addition, tannic acid treatment significantly decreased the GST activity in the midgut of *H. cunea* larvae, suggesting that tannic acid could inhibit the detoxification ability of larvae and aggravate the toxicity due to tannic acid. Our results were in line with those reported by Chen et al., who showed that tannic acid treatment decreased GST activity in *H. armigera* and slowed down the processes of growth and development [49].

Accumulating evidence shows that xenogenous toxicants may change the composition of the gut microflora, thus destroying the normal physiological functions of the gut microbiota and adversely affecting the growth and development of insects [50,51]. In this study, tannic acid treatment was found to significantly affect the richness and taxonomic composition of larval gut microbiota at the experimental dose. The Chao1 index of gut microbes increased significantly in the tannin-treated group, which suggested that tannic acid increased the species richness of gut microbiota in the *H. cunea* larvae. However, tannic acid exerted no substantial effects on the Shannon index of gut microflora, which indicated that the community diversity did not alter significantly. In addition, from the perspective of taxonomic composition, tannic acid exposure significantly altered the changes in the gut microbial community of the larvae. At the phylum level, there was little difference in the intestinal microbial composition of the control/treatment groups, and proteobacteria dominated the intestinal microbial composition. However, tannic acid treatment significantly altered the abundances of the gut microbiota at the genus level in the *H. cunea* larvae, as evidenced by the fact that the dominant genus in the tannin-treated group was significantly different from that of the control group.

To identify the differences in the gut microbiota at the genus level in *H. cunea* larvae upon tannic acid exposure, we performed an LEfSe analysis for the microbiota. The results showed that, at the genus level, the abundances of *Brevundimonas*, *Delftia*, *Sphingomonas*, *Novosphingobium*, *Dechloromonas*, *Methyloversatilis*, and *Devosia* in the tannin-treated group were significantly lower than those in the untreated group. Among these, *Devosia* can degrade starch, and *Dechloromonas* is a type of denitrifying bacteria [52,53]. The decline in their abundances indicated that tannic acid decreased the nutrient degradation ability in *H. cunea* larvae. *Brevundimonas* and *Novosphingobium* are involved in the degradation of cellulose and lignin, respectively, and a reduction in their abundance may lead to the inhibition of degradation and metabolism of plant secondary metabolites in the tannin-treated *H. cunea* larvae [54,55]. *Delftia*, *Sphingomonas,* and *Methyloversatilis* bacteria can resist toxic substances such as insecticides and antibiotics [56,57,58]. A decrease in the abundance of these microbial genera indicated that tannic acid treatment inhibited the defense ability of the larvae against xenogenous toxins. The prediction of functions using PICRUSt2 confirmed the above inferences, and we found that the functions of gut microbiota related to nutritional metabolism (e.g., amino acids, carbohydrate or lipid transport, and metabolism) and detoxification metabolism (e.g., secondary metabolites biosynthesis, transport, and catabolism) were significantly suppressed in the tannin-treated group. The downregulation of these pathways was consistent with the decrease in nutrient absorption and metabolism, as well as a reduction in the microbiota related to nutrition and xenogenous toxin metabolism in *H. cunea* larvae under tannic acid stress, further highlighting the detrimental effects of tannic acid on larval nutrition and detoxification metabolism. The alterations in gut microbial abundances and diversity not only directly affect the functions of microbiota but also indirectly change the physiological state of the larvae [59]. Our correlational analysis for the gut microbiota and physiological parameters supported this deduction and indicated that disordered gut microflora may increase the toxic effects of tannic acid on *H. cunea* larvae at multiple levels.

## 5. Conclusions

Combined with the biochemical parameters and gut microbiota, this study reveals the toxic effects of tannic acid and its mechanisms of action against *H. cunea* larvae. Oxidative damage to the larval gut of *H. cunea* is a major toxic effect of tannic acid. This results in decreased food utilization efficiency and gut microbiota disorder in larvae of *H. cunea*. The disturbance of gut function is the main reason that *H. cunea* larvae can not grow normally or survive to pupate under tannic acid treatment. Tannic acid is a potential botanical pesticide for the control of *H. cunea* larvae.

## Figures and Tables

**Table 1:** The cumulative mortality rate, pupation rate, and emergence rate of *H. cunea* larvae after rearing on untreated or tannic acid-treated artificial diets.

		Control	Treatment (12.5 mg/g)
Larval mortality rate	4th instar	0.00 ± 0.00% a	6.67 ± 1.76% b
	5th instar	0.00 ± 0.00% a	19.33 ± 2.91% b
	6th instar	0.00 ± 0.00% a	45.67 ± 4.37% b
	Mature larvae	0.00 ± 0.00% a	100.00 ± 0.00% b
Pupation rate Emergence rate		70.00 ± 5.78% a	0.00 ± 0.00% b
		80.95 ± 6.67% a	0.00 ± 0.00% b

The cumulative mortality rate of each instar is calculated at the beginning of this instar. Different lowercase letters indicate significant differences between control/treatment groups (*n* = 3; independent samples *t*-test; *p* < 0.05).

## Data Availability

The data presented in this study are available on request from the corresponding author.

## Supplementary Materials

The following supporting information can be downloaded at: https://www.mdpi.com/article/10.3390/insects13100872/s1, Figure S1: Venn diagram for operational taxonomic units in the control and treatment groups; Figure S2: Proportion of bacteria at the phylum (A) and genus (B) level in the *H. cunea* larvae at the 6th instar after rearing on un-treated or tannic acid-treated diets.; Figure S3: Linear discriminant analysis effect size (LEfSe) cladogram of gut microbiota in the *H. cunea* larvae at the 6th instar after rearing on untreated or tannic acid-treated diets (A). Nodes that are not yellow indicate a significant contribution to grouping. (B) LDA values of the distribution histogram.
