# Supplementary material for: Evaluating the Toxic Effects of Tannic Acid Treatment on Hyphantria cunea Larvae
Source: Insects. 2022 Sep 26;13(10):872. doi: 10.3390/insects13100872 (PMC9604457; doi:10.3390/insects13100872)
Supplement: Supplementary file 1 [file insects-13-00872-s001.zip › insects-1891258-supplementary.pdf]

Supplementary materials

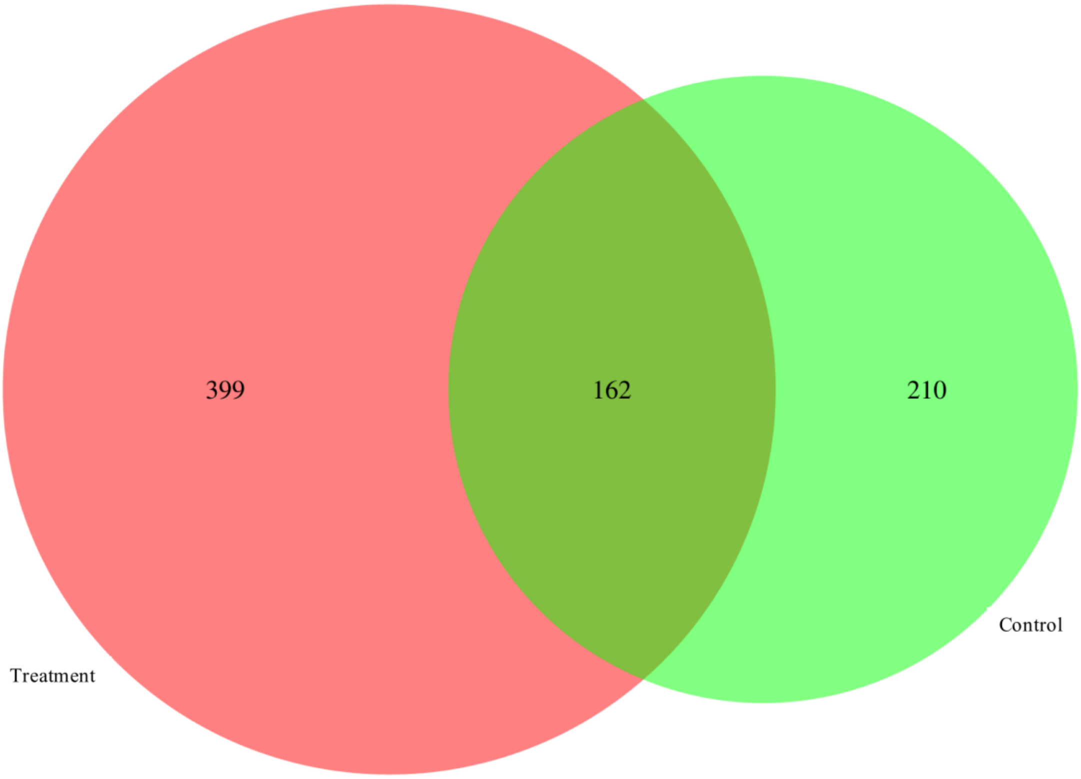

Figure S1. Venn diagram for operational taxonomic units in the control and treatment groups.

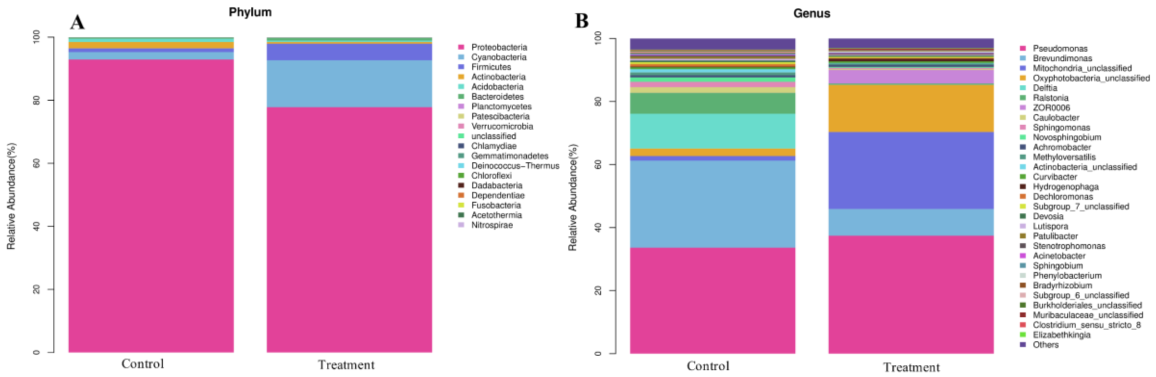

Figure S2. Proportion of bacteria at the phylum (A) and genus (B) level in the *H. cunea* larvae at the 6th instar after rearing on un-treated or tannic acid-treated diets.

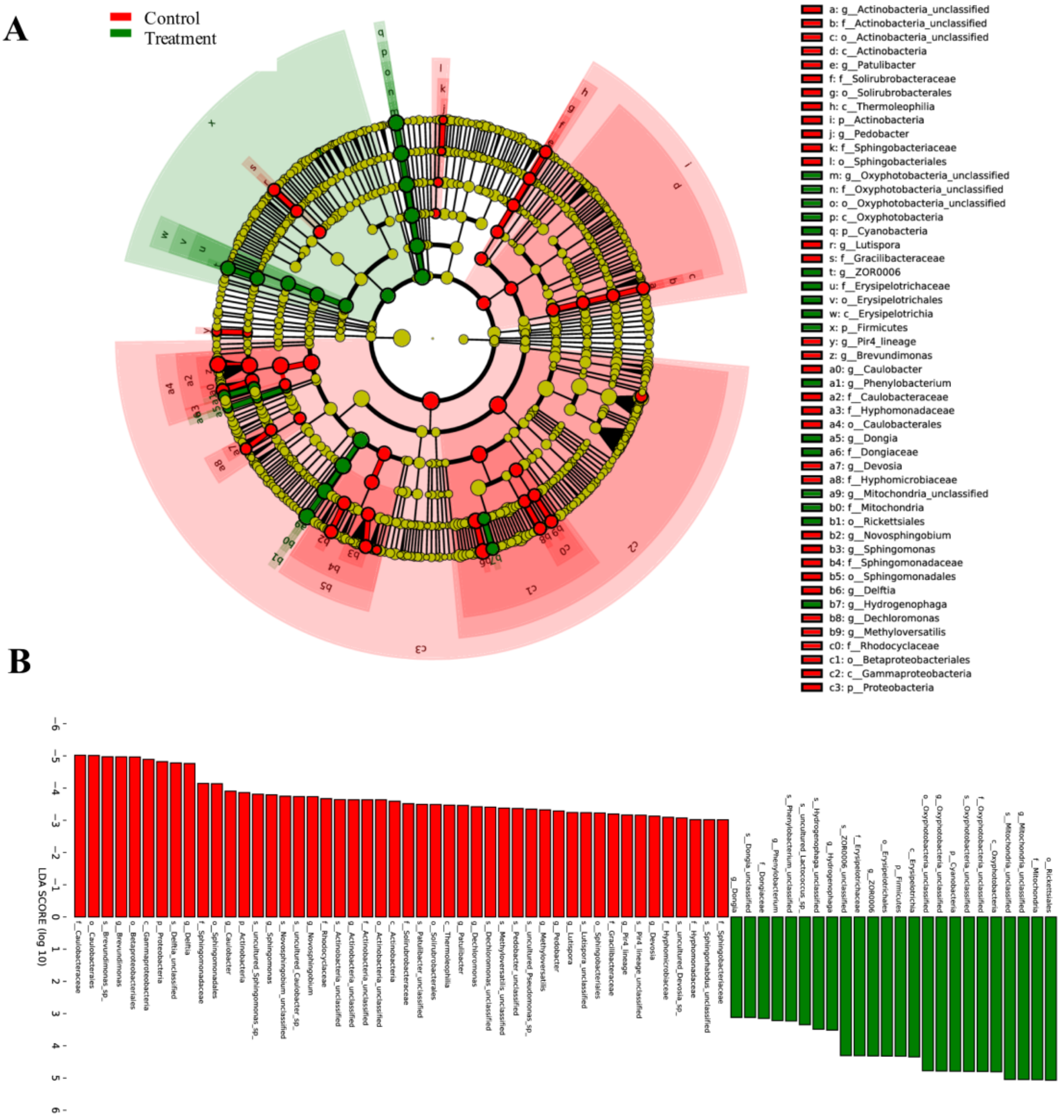

Figure S3. Linear discriminant analysis effect size (LEfSe) cladogram of gut microbiota in the *H. cunea* larvae at the 6th instar after rearing on untreated or tannic acid-treated diets (A). Nodes that are not yellow indicate a significant contribution to grouping. (B) LDA values of the distribution histogram.
